# Using Smartphone Sensor Data to Assess Inhibitory Control in the Wild: Longitudinal Study

**DOI:** 10.2196/21703

**Published:** 2020-12-04

**Authors:** Vincent WS Tseng, Jean Dos Reis Costa, Malte F Jung, Tanzeem Choudhury

**Affiliations:** 1 Department of Information Science Cornell University New York, NY United States; 2 DawnLight Technologies Palo Alto, CA United States; 3 Department of Information Science Cornell University Ithaca, NY United States

**Keywords:** self-control, mobile phone

## Abstract

**Background:**

Inhibitory control, or inhibition, is one of the core executive functions of humans. It contributes to our attention, performance, and physical and mental well-being. Our inhibitory control is modulated by various factors and therefore fluctuates over time. Being able to continuously and unobtrusively assess our inhibitory control and understand the mediating factors may allow us to design intelligent systems that help manage our inhibitory control and ultimately our well-being.

**Objective:**

The aim of this study is to investigate whether we can assess individuals’ inhibitory control using an unobtrusive and scalable approach to identify digital markers that are predictive of changes in inhibitory control.

**Methods:**

We developed InhibiSense, an app that passively collects the following information: users’ behaviors based on their phone use and sensor data, the ground truths of their inhibition control measured with stop-signal tasks (SSTs) and ecological momentary assessments (EMAs), and heart rate information transmitted from a wearable heart rate monitor (Polar H10). We conducted a 4-week in-the-wild study, where participants were asked to install InhibiSense on their phone and wear a Polar H10. We used generalized estimating equation (GEE) and gradient boosting tree models fitted with features extracted from participants’ phone use and sensor data to predict their stop-signal reaction time (SSRT), an objective metric used to measure an individual’s inhibitory control, and identify the predictive digital markers.

**Results:**

A total of 12 participants completed the study, and 2189 EMAs and SST responses were collected. The results from the GEE models suggest that the top digital markers positively associated with an individual’s SSRT include phone use burstiness (*P*=.005), the mean duration between 2 consecutive phone use sessions (*P*=.02), the change rate of battery level when the phone was not charged (*P*=.04), and the frequency of incoming calls (*P*=.03). The top digital markers negatively associated with SSRT include the standard deviation of acceleration (*P*<.001), the frequency of short phone use sessions (*P*<.001), the mean duration of incoming calls (*P*<.001), the mean decibel level of ambient noise (*P*=.007), and the percentage of time in which the phone was connected to the internet through a mobile network (*P*=.001). No significant correlation between the participants’ objective and subjective measurement of inhibitory control was found.

**Conclusions:**

We identified phone-based digital markers that were predictive of changes in inhibitory control and how they were positively or negatively associated with a person’s inhibitory control. The results of this study corroborate the findings of previous studies, which suggest that inhibitory control can be assessed continuously and unobtrusively in the wild. We discussed some potential applications of the system and how technological interventions can be designed to help manage inhibitory control.

## Introduction

### Background

Inhibitory control, or inhibition, is the ability to inhibit prepotent responses to goal-irrelevant stimuli. It is one of our executive functions and is essential for sustained attention [1], working memory [2], and emotion regulation [3], which in turn contribute to our performance and well-being. Studies have shown that students who demonstrate better inhibitory control tend to be better at time management and achieve higher academic performance [4-9] and that employees who have higher inhibitory control are more likely to have higher motivation and productivity [10]. On the other hand, reduced inhibitory control can lead to attention problems and impulsive and addictive behaviors [11] (eg, alcohol and drug addiction) [12]. Furthermore, many mental disorders are associated with impaired inhibitory control, including eating disorders [13], posttraumatic stress disorder [14], bipolar disorder [15], and schizophrenia [16].

Although some individuals have lower inhibitory control than others, our inhibitory control is not a stable trait. Instead, it fluctuates over time based on internal and environmental factors, such as our moods, activities, and the context or the surrounding environment we are in. For example, it has been shown that physical activities [17] and good sleep habits [18] have positive effects on inhibitory control. Furthermore, exposure to natural landscapes can enhance one’s inhibitory control. People who were exposed to natural landscapes showed a higher ability for future valuation (ie, an individual's willingness to wait for a longer period of time in exchange for a larger reward) and delayed gratification compared with those exposed to urban environments [19]. Moreover, even just showing participants images of natural environments could significantly reduce their impulsive decision making compared with showing them images of human-made environments or geometric shapes [20].

As the ebbs and flows of inhibitory control consequently influence an individual’s daily behaviors, such as attentional ability [21], alcohol consumption [22,23], and antisocial and criminal behaviors [24], researchers have examined different approaches to assessing inhibitory control, including psychometric tests, physiological signals, and self-report questionnaires. For example, stop-signal task (SST) [25] is one of the most widely used psychometric tests; it measures participants’ reaction time to inhibit their prepotent response after seeing a stop signal. It has been used to predict the level of smokers’ craving for cigarettes [26]. Physiological signals, such as heart rate variability (HRV), also have been shown to be indicators of the level of an individual’s inhibitory control. Individuals with higher HRV tend to have higher inhibitory control, as HRV is associated with one's ability to adjust in response to changes in the environment [27]. A number of self-control scales were used in studies that examined how people's self-control contributes to regular exercise [28] and web game addiction [29]. However, most of the studies were conducted in laboratory settings and only focused on the inhibitory control of individuals with cravings or addiction. To measure inhibitory control in the wild, asking users to periodically complete psychometric tests or self-report questionnaires can be burdensome, as each session of psychometric tests normally takes about 5 min. Moreover, self-reported responses are likely to be biased by responders’ current inhibitory control [30,31]. Thus, assessing inhibitory control using self-reports may be subject to subjectivity and unreliability.

Some of the aforementioned factors that modulate inhibitory control, such as locations visited and physical activities, can be captured with smartphones or wearables. Behavioral patterns that are associated with changes in inhibitory control may also manifest themselves in people's phone sensor data, such as their phone use patterns. Some previous work suggested that phone sensor data can be used to predict an individual's cognitive performance [32]. However, the relationship between an individual's inhibitory control and behavioral patterns has yet to be fully studied. HRV can also be collected continuously in the wild using a chest strap or a wristband. However, the reliability of phone sensor data, particularly for the purpose of assessing inhibitory control, has not been fully investigated so far.

### Objectives

To this end, the goal of this study is to investigate measuring inhibitory control using a scalable approach within a broader population in addition to people who have problems with inhibitory control and markers indicative of changes in inhibitory control. We developed an iOS app and conducted a 4-week in-the-wild study to collect users’ phone sensor data, HRV, and subjective and objective measurements of their inhibitory control. We used the data to infer the changes in participants’ inhibitory control and to examine the factors that were associated with the changes. The contributions of this paper are 3-fold:

1. To the best of our knowledge, this is the first in-the-wild study that collected participants’ inhibitory control with objective and subjective measurements along with their phone sensor data and HRV. We also made our code for our InhibiSense publicly available on Github [33] so that other researchers can use the platform to conduct future studies.
2. We identified how different behaviors and contexts influenced people’s inhibitory control in the wild based on more than 1100 measurements collected from this study. We also used these predictors as features to train machine learning models to determine whether we could predict high and low inhibitory control.
3. Finally, we discussed the implications of the study and several applications of the system that can help us manage our behaviors and well-being in real-world scenarios.

## Methods

### System

**Figure 1:**
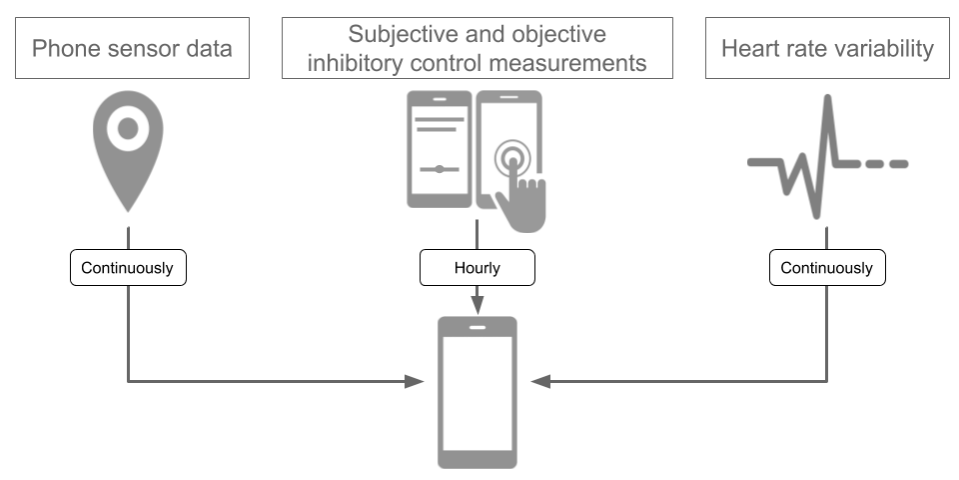
Data collected on participants' smartphones.

We developed InhibiSense, an iOS study app that collects participants’ phone sensor data, HRV, ecological momentary assessment (EMA), and performance of SSTs (Figure 1). The collected data were temporarily stored on a participant’s phone and uploaded to our server when the participant’s phone was charged and connected to the internet through a Wi-Fi network. The details of each data type are described below.

#### Phone Sensor Data

InhibiSense collected users’ phone sensor data continuously using the AWARE framework [34]. The AWARE framework provides application programming interfaces (APIs) that allow researchers to integrate phone sensor data logging and storing into their study apps. Specifically, the types of data we collected include participants’ GPS locations, Wi-Fi device names (service set identifier [SSID]), activities, ambient noise, networks, phone use, calls, battery status, and time.

#### HRV

HRV calculation is based on a person’s interbeat intervals, or RR intervals. We collected participants’ RR intervals using Polar H10 [35]. The RR intervals’ recordings were transmitted to InhibiSense via Bluetooth.

#### EMA

EMA is a method to repeatedly sample participants’ experiences in real time during a study [36]. We employed a valid EMA for inhibitory control that was suited for the context of our research [28]. It consisted of 6 questions regarding one’s inhibitory control at the present moment, such as “I have to force myself to stay focused*”* (Table 1). Participants were prompted to select a score for each question on a Likert scale ranging from 0 (*Not at all)* to 6 (*Very much so*) using a slider.

**Table 1:** Types of data collected during the study.

Type	Description	Frequency
Demographic information	Barratt Impulsiveness Scale Sex Age	During the onboarding meeting
Phone sensor	Physical activity Phone use Call Battery level GPS location Wi-Fi service set identifier Ambient noise Network Time	Continuously
Heart rate variability	Interbeat interval	Continuously
Ecological momentary assessment	Six self-report questions asking about one’s inhibitory control at the moment, including the following: “I have to force myself to stay focused” “I am full of willpower” “I am having trouble pulling myself together” “I could resist any temptation” “I am having trouble paying attention” “I have no trouble bringing myself to do disagreeable things”	Prompted every hour from 7 AM to 11 PM
stop-signal task	Each session consisted of 80 trials, 60 of which were Go trials and 20 of which were stop trials. The order of the trials was randomized. A tracking method was used where the SSD^a^ was increased by 25 milliseconds if a stop error was made and decreased by 25 milliseconds if a stop was successful.	Prompted every hour from 7 AM to 11 PM following the completion of the ecological momentary assessment

^a^SSD: stop-signal delay.

#### SST

SSTs are valid and widely used objective measurements for assessing inhibitory control [37]. They typically consist of 2 types of signals: go signals and stop signals. stop signals appear at some random intervals, which are often referred to as stop-signal delays (SSDs), after go signals with a predetermined probability. Participants have to respond to go signals as quickly as they can and inhibit their response when they see a stop signal.

**Figure 2:**
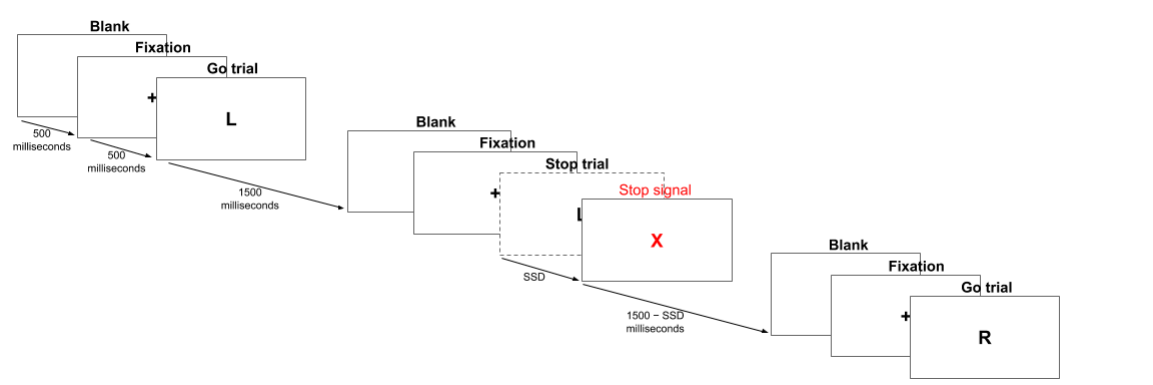
Illustration of the stop-signal task implementation. During the task, Go trials and stop trials appeared in random orders. During a Go trial, a Go stimulus, either a letter "L" or a letter "R" was displayed, to which a participant had to respond. During a stop trial, a Go stimulus was first displayed and then followed by a stop signal "X" after a certain stop Signal Delay, in which a participant had to inhibit their response. At the beginning of each trial, the screen turned blank for 500ms followed by 500ms fixation duration with a "+" displayed at the center of the screen. SSD: stop-signal delay; SST: stop-signal task.

Our implementation of SST (Figure 2) consisted of 80 trials, 75% (60/80) of which were go trials and the rest were stop trials. The order of the trials was randomized during each session. Having a higher probability of showing a go signal is a recommended approach to preventing participants from strategically delaying their response and waiting for a stop signal. The interval of each trial was 1500 milliseconds. The stimuli disappeared immediately when a participant touched the screen. In each go trial, a letter *L* or *R* was shown with equal probability, and a participant had to tap the left or the right side of the screen, respectively. In a stop trial, the go signal was replaced with a stop signal *X* after an SSD, and a participant had to inhibit their response immediately. To estimate a participant's stop-signal reaction time (SSRT) more accurately, we used the tracking method (the one-up-one-down method). The initial SSD was set to 250 milliseconds and then was increased by 25 milliseconds in the next stop trial after a successful stopping and was decreased by 25 milliseconds after an unsuccessful stopping.

### Study

The study lasted for 4 weeks. During the study, participants received push notifications for answering the EMA questions and completing the SST every hour, from 7 AM to 11 PM. Each task would expire in an hour. They were encouraged to wear the heart rate monitor for the entire day and were able to proceed with the task only if they were wearing it.

### Recruitment

The study was approved by the Institutional Review Board at Cornell University. We used convenience sampling for our recruitment. During the onboarding meeting, participants were asked to fill out a demographic questionnaire, including their age range and sex, and a Barratt Impulsiveness Scale (BIS 11) questionnaire [38]. Then, they were informed of the flow of the study and signed a consent form. Each participant was given a Polar H10 to wear throughout the study. Finally, the participants installed InhibiSense on their phones and were instructed to complete a couple of practice sessions for SST to ensure that they fully understood the process. Participants were given compensation at the end of the study based on the number of sessions (US $1 for each session) they had completed.

### Data Preprocessing and Feature Extraction

Our goal was to assess an individual’s inhibitory control at a more granular level, specifically every hour. As such, we needed to use the information on the context and physiological signals during the past hour to infer a participant’s inhibitory control at the end of the 1-hour window. We extracted passive sensing features from the phone sensor data and HRV features from their RR intervals during the 1-hour window before a participant started an SST. Specifically, the features from phone sensor data capture information on factors that are associated with changes in inhibitory control, including activities, phone use, surrounding environments, and sleep. We will describe our data preprocessing and feature extraction below.

#### Individual Inhibitory Control Baseline

The questions asked in BIS correspond to different order factors (dimensions). Specifically, 6 out of the total 30 questions correspond to order factor *self-control*, also known as trait inhibitory control. We added up the scores for the 6 questions and used it as the baseline inhibitory control.

#### Phone Sensor Features

##### Activity

Activity can be broken down into the following categories: total number of steps, distance traveled, activity type, and acceleration. We computed the percentage of time an individual was stationary, walking, running, automotive, cycling, and nonstationary (which includes all the activities except for being stationary) during each 1-hour window based on the inferred activities provided by Apple’s activity recognition API [39]. For acceleration, we computed the mean and standard deviation of a user’s acceleration (m/s^2^).

##### Phone Use

We computed phone use–related features as follows:

1. Phone use sessions: On the basis of screen unlock and lock events, we computed the burstiness (the number of phone use sessions), the number of short sessions (sessions less than 30 seconds) [40], the total duration of phone use, the mean and standard deviation of phone use duration, and the intervals between consecutive phone use sessions.
2. Call: The number and the mean duration of incoming and outgoing calls.
3. Battery: The frequency and percentage of time a user’s phone was being charged and the rate of battery level change when the phone was and was not being charged, respectively.

##### Environment

We computed environment-related features as follows:

1. Ambient noise: We computed the mean, median, and standard deviation of the frequency, loudness (dB), and power, namely root mean square, of ambient noise.
2. Location: We used the GPS coordinates and Foursquare API [41] to retrieve the semantic locations, namely, the categories of the places a participant visited. As the GPS locations might sometimes be inaccurate, we obtained the categories of a user’s nearby locations within 50 m for each GPS location and aggregated all the distinct location categories within each window. As we were interested in investigating the relationship between the consistency of environments and a person’s inhibitory control, we also retrieved the locations that a participant had been to in the previous window (ie, between 1 and 2 hours before the start of an SST) and computed the similarity of the locations visited between the 2 consecutive windows using the Jaccard, Dice, Second-Kulczynski, and Ochiai distance metrics [42-45].
3. Wi-Fi: We computed the similarity between the Wi-Fi device names (SSID) in 2 consecutive windows using the Jaccard, Dice, Second_Kulczynski, and Ochiai distance metrics. The presence of the individual SSIDs during a window was encoded as a binary vector, and the similarity metrics were then applied to the vectors.
4. Network: The percentage of time a user's phone was connected to the internet and the percentage of time the phone was connected to the network through a mobile and Wi-Fi network, respectively.

##### Sleep

Sleep duration, sleep onset, and sleep offset were inferred based on participants’ phone use [46]. Sleep duration was clipped to 2 standard deviations from the mean to replace the outliers.

##### Time

Information on the day of the week, weekday or weekend, and the hour of the day was obtained based on the timestamps.

The features extracted for each type of sensor data are summarized in Textbox 1.

**Features extracted from the sensor data.**

Physical activity

- Mean and standard deviation of acceleration
- Number of steps
- Distance traveled
- Percentage of time a participant was stationary, walking, running, automotive, cycling

Phone use

- Phone use burstiness
- Frequency of short phone use sessions
- Mean and standard deviation of phone use durations (seconds) and intervals between consecutive phone use sessions

Call

- Frequency and duration (seconds) of incoming and outgoing calls

Battery

- Frequency and duration (seconds) of charging
- Mean change rate of battery level (units/second) when the phone was charged and not charged

GPS location

- Location category
- Jaccard, Dice, Second_Kulczynski, and Ochiai coefficients of location similarity

Wi-Fi

- Jaccard, Dice, Second_Kulczynski, and Ochiai coefficients for Wi-Fi service set identifier (SSID) similarity

Ambient noise

- Mean and standard deviation of frequency (Hz), loudness (dB), and sound root mean square (RMS)

Network

- Percentage of time the phone was connected to the internet through a Wi-Fi or mobile network or was not connected

Time

- Weekend or weekday
- Day of the week
- Hour of the day

Sleep

- Sleep onset and offset
- Hours of sleep

Interbeat interval

- Mean and standard deviation of heart rate
- Standard deviation of NN intervals
- Standard deviation of 5-min average NN intervals
- Root-mean-squared NN interval differences
- Standard deviation of NN interval differences
- Triangular index
- Relative power of low (0.04-0.15 Hz) and high (0.15-0.4 Hz) frequency band computed using Fast Fourier transform (FFT), Lomb-Scargle periodogram, and autoregressive method

#### HRV Features

To ensure the quality of the HRV features, HRV features were extracted if and only if an interval contained at least 5 min of continuous RR interval recordings. For windows that met the criteria, we first removed outlier and ectopic beats [47] and replaced them using cubic interpolation using the HRVanalysis package [48]. Then, the time-domain, frequency-domain, and nonlinear features were extracted using the pyHRV package [49] (Textbox 1).

#### SST Performance Metrics

SSRT is the amount of time it takes for a participant to inhibit their response, which is inversely correlated with one’s inhibitory control. A longer SSRT means lower inhibitory control and vice versa. We used the integration method based on the horse race theorem [25] to compute the central SSRT, the most reliable way to estimate SSRT, in each session. For each SSD in a given session, we computed the stop unsuccessful rate p(response | signal) and the corresponding *n*th RT (the RT at the *n*th percentile) among the RT distribution in that session, where n is equal to (p(response | signal)×100). Then, we used linear interpolation to find the SSD where p(response | signal) equals 0.5. Finally, the central SSRT in that session was obtained by subtracting the SSD from the 50th RT.

### Outlier Exclusion

We excluded data points in which the stop unsuccessful rate was below 0.25 or above 0.75 and the estimated SSRT was below 50 milliseconds or above 1500 milliseconds [50].

### Data Analysis

#### The Relationship Between Subjective and Objective Measurements of Inhibitory Control

To investigate how the different constructs of inhibitory control in the EMA are related to each other and how people perceive their own inhibitory control in comparison with their inhibitory control measured using SST, we computed the correlation coefficients between the different dependent variables using repeated measures correlation (RMCORR) [51,52]. Repeated measures correlation is used to measure the strength of the relationship between 2 variables in repeated measurements across different participants, which accounts for the intraindividual associates between 2 measures.

#### Features Predictive of Changes in Inhibitory Control

To examine the predictive features for inhibitory control, namely, features that have significant main effects for estimating SSRT, we fitted 2 generalized estimating equation (GEE) [53] models with 2 different feature sets, one with only phone sensor features and the other with phone sensor features along with HRV features, to predict participants’ SSRT. GEE is a statistical model that is used to identify feature variables that have significant effects during repeated measurements and in the meantime to account for individual differences, which is particularly useful for analyzing the relationship between the predictors and outcomes in longitudinal studies. Developing models with these 2 feature sets helped us get a better understanding of how models perform using the least obtrusive manner, namely, by using only phone sensor data, and whether the models’ performance will improve when the information on HRV is accessible and incorporated.

In our analysis, the independent variables were the sensor features and their inhibitory control baseline derived from their BIS responses; the dependent variable was SSRT. We chose SSRT to be the dependent variable because we assumed that an individual's inhibitory control is a continuum. Before fitting the models, we first removed features whose collinearity was above the variance inflation factor threshold [54] and then used the remaining features as the independent variables for the models. Gamma distribution was used in the GEE models to model the distribution of SSRT.

#### Predicting States of Inhibitory Control

After determining which features are more predictive of changes in inhibitory control, our next research question was whether we can automatically infer the state of a person’s inhibitory control using these features. To this end, we trained gradient boosting tree (GBT) [55] classifiers to classify whether an individual was in a high or low inhibitory control state with both feature sets (phone sensor features only and phone sensor features plus HRV features) after the predictive features were identified. A high or low inhibitory control state refers to whether an individual’s SSRT during an SST session was lower or higher than their overall median SSRT. GBT is a type of classifier that is more robust to outliers. The features were z-standardized before fitting into the classifiers. We used leave-one-subject-out cross-validation, where 1 participant’s data were held out for testing and a model was trained on the remaining participants’ data during each iteration, to evaluate the model performance. The metrics for the cross-validation are the mean accuracy and the area under the receiver operating characteristic curve (AUC-ROC) [56].

## Results

### Descriptive Statistics

**Figure 3:**
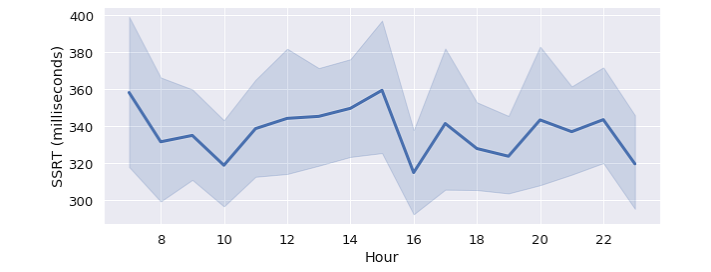
Mean stop-signal reaction time at different times of day. The shaded area indicates 95% CIs. SSRT: stop-signal reaction time.

A total of 22 participants signed up for the study; 10 of them dropped out in the middle of the study due to their school workloads. The remaining participants (their demographics are summarized in Table 2) completed 2189 tasks over the course of the study. After applying data preprocessing and outlier exclusion, data from 1107 sessions (mean 92.3, SD 67.4) were used for our analysis. Table 3 shows the cumulative number of tasks completed during the different hours; the data were roughly uniformly distributed across 9 AM to 11 PM. Figure 3 shows the mean SSRT at different times of day averaged across all the participants. Overall, participants had lower SSRT around 10 AM and 4 PM and higher SSRT around 7 AM and 3 PM; however, no statistically significant difference was found.

**Table 2:** Demographics of the participants (N=12).

Variable		Participants, n (%)
**Sex**		
	Female	10 (83)
	Male	2 (17)
**Age (years)**		
	18-22	7 (58)
	23-27	2 (17)
	28-32	2 (17)
	38-42	1 (8)

**Table 3:** The cumulative numbers of completed tasks at different times of day N=1107.

Time (hours)	Count, n (%)
7	26 (2)
8	34 (3)
9	55 (5)
10	61 (6)
11	65 (6)
12	66 (6)
13	82 (7)
14	68 (6)
15	80 (7)
16	84 (8)
17	73 (7)
18	65 (6)
19	73 (7)
20	64 (6)
21	76 (7)
22	73 (7)
23	60 (5)

**Figure 4:**
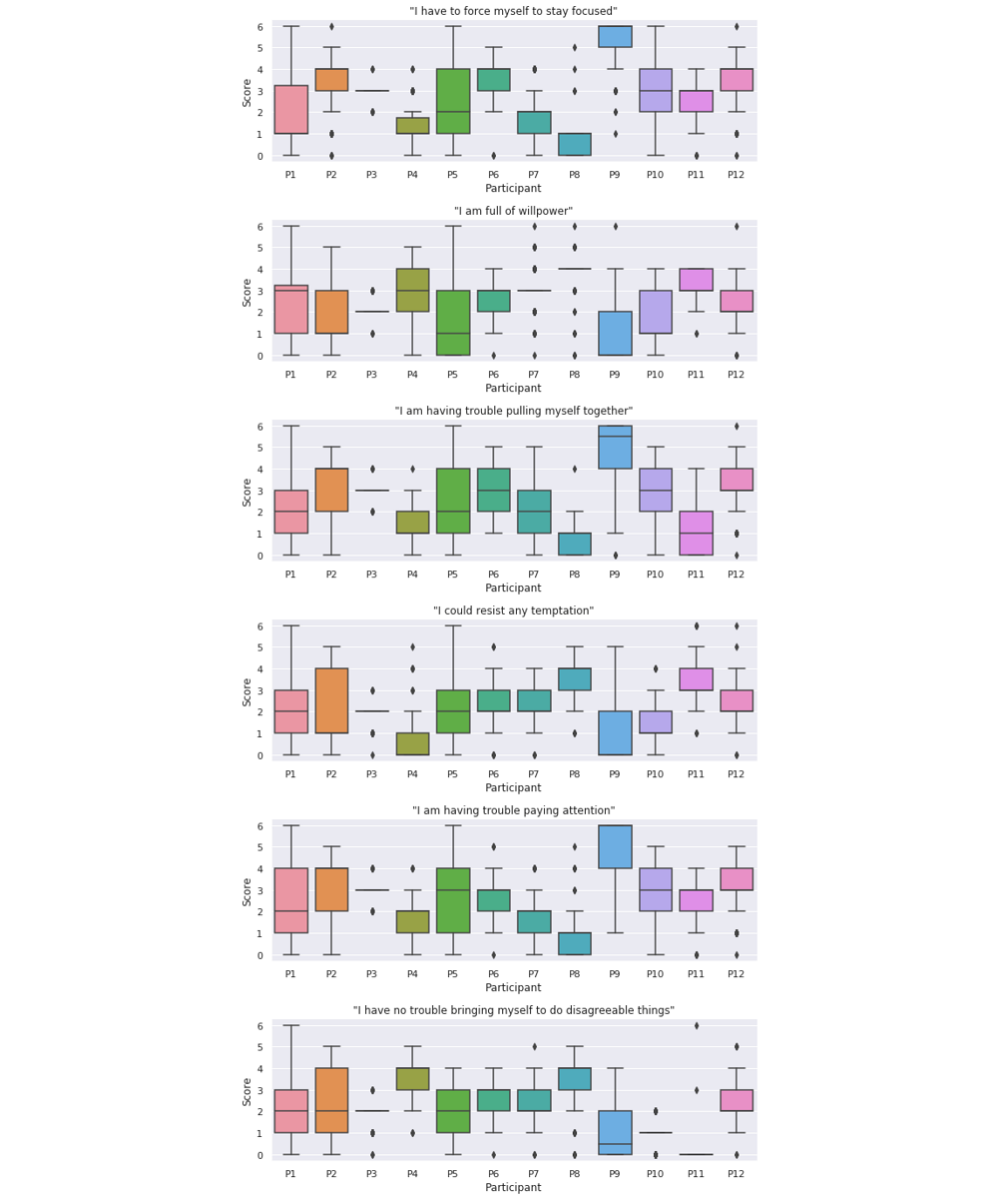
Distribution of the responses for the individual ecological momentary assessment questions. EMA: ecological momentary assessment.

**Figure 5:**
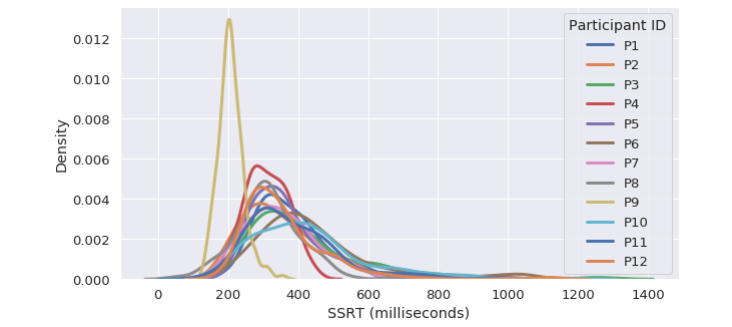
Distribution of participants’ stop-signal reaction time. SSRT: stop-signal reaction time.

**Figure 6:**
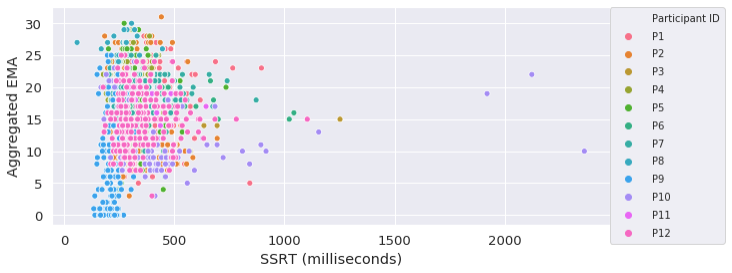
Relationship between participants’ aggregated ecological momentary assessment scores and stop-signal reaction times. EMA: ecological momentary assessment; SSRT: stop-signal reaction times.

The distribution of participants’ responses to the individual EMA questions and their SSRT are shown in Figures 4 and 5, respectively. Participant P9 had an overall shorter SSRT compared with the other participants. In the meantime, this participant also had a higher average stop unsuccessful rate. We found that all the EMA questions had strong RMCORR with each other (all the *P* values were <.001). Conversely, none of the EMA questions had significant RMCORR with SSRT, except that the responses for the EMA question “I am having trouble pulling myself together” had a marginal positive correlation with SSRT (RMCORR=0.041; 95% CI −0.01 to 0.09; *P*=.09). No statistically significant RMCORR between the aggregated EMA score (the scores for the negatively worded questions were first inverted before being added together) [28] and SSRT was found. Figure 6 shows the relationship between participants’ aggregated EMA scores and their SSRTs.

### Features Predictive of Changes in Inhibitory Control

Table 4 shows the coefficients and their corresponding confidence intervals of the individual phone sensor features for predicting SSRT estimated by the GEE model. The features that had statistically significant effects on predicting SSRT include an individual’s internal factors and factors associated with their activity, phone use, and the surrounding environment. It is worth nothing that an individual’s SSRT and inhibitory control are inversely related. In other words, a higher SSRT implies a lower inhibitory control, and vice versa.

**Table 4:** The coefficients for all the phone sensor features for predicting stop-signal reaction time estimated by the generalized estimating equation model. A positive coefficient implies that the feature is associated with higher stop-signal reaction time (lower inhibitory control).

Feature	Estimated coefficient (95% CI)	*P* value
Individual inhibitory control baseline	1.04 (0.42 to 1.66)	.001
Phone use burstiness	0.0213 (0.006 to 0.036)	.005
Mean duration between 2 consecutive phone use sessions	4.791e-08 (8.11e-09 to 8.77e-08)	.02
Frequency of short phone use sessions	−0.0204 (−0.028 to −0.012)	<.001
Change rate of battery level when the phone was not charged	0.0479 (0.002 to 0.093)	.04
Frequency of incoming calls	0.1333 (0.011 to 0.256)	.03
Mean duration of incoming calls	−0.0006 (−0.001 to 0.000)	<.001
Standard deviation of acceleration	−0.4009 (−0.624 to −0.177)	<.001
Standard deviation of ambient noise frequency	−3.811e-05 (−7.04e-05 to −5.83e-06)	.02
Mean decibel level of ambient noise	−0.5267 (−0.912 to −0.141)	.007
Percentage of time connected to the internet through a mobile network	−0.1717 (−0.270 to −0.074)	.001
Jaccard coefficient of location similarity	−0.0415 (−0.078 to −0.005)	.03
In outdoor or recreational locations during the hour before the past hour	−0.0682 (−0.120 to −0.016)	.01
In residential buildings during the hour before the past hour	0.0719 (0.001 to 0.142)	.045
In residential buildings during the past hour	0.1185 (0.002 to 0.235)	.046
Mean change rate of battery level when the phone was charged	−0.0074 (−0.016 to 0.093)	.08
Frequency of charging	0.0116 (−0.037 to 0.060)	.64
Total duration of charging	1.084e-05 (−2.26e-05 to 4.43e-05)	.53
Hours of sleep	−0.0102 (−0.023 to 0.003)	.12
Mean phone use duration	3.971e-08 (−6.78e-09 to 8.62e-08)	.09
Standard deviation of phone use durations	3.536e-08 (−3.29e-08 to 1.04e-07)	.31
Standard deviation of durations between 2 consecutive screen unlocks	6.596e-08 (−2.11e-08 to 1.53e-07)	.14
Frequency of outgoing calls	0.0151 (−0.026 to 0.056)	.47
Total duration of outgoing calls	−6.287e-05 (−0.001 to 0)	.50
Percentage of time being stationary	−0.1276 (−0.275 to 0.020)	.09
Standard deviation of the decibel level of ambient noise	0.0282 (−0.294 to 0.351)	.86
Mean RMS^a^ level of ambient noise	0.0003 (−0.001 to 0.001)	.50
Standard deviation of the RMS level of ambient noise	−0.0002 (−0.002 to 0.001)	.77
In locations for arts or entertainment during the past hour	−0.0259 (−0.157 to 0.105)	.70
In professional or other places during the past hour	0.1102 (−0.028 to 0.248)	.12

^a^RMS: root mean square.

Specifically, in terms of internal factors, individual inhibitory control baseline had a significant positive effect on predicting SSRT (95% CI 0.421-1.662; *P*=.001), that is, lower trait inhibitory control (higher self-reported score in BIS) was associated with higher SSRT (lower inhibitory control). Regarding activity, the standard deviation of acceleration had a significant negative effect (95% CI −0.624 to −0.177; *P*<.001); larger changes in one’s movement were associated with higher inhibitory control. As for phone use, phone use burstiness (95% CI 0.006-0.036; *P*=.005), the mean duration between 2 consecutive phone use sessions (95% CI 8.11e-09-8.77e-08; *P*=.02), the change rate of battery level when the phone was not charged (95% CI 0.002-0.093; *P*=.04), and the frequency of incoming calls (95% CI 0.011-0.256; *P*=.03) had significant positive effects on predicting SSRT (associated with lower inhibitory control), whereas the frequency of short phone use sessions (95% CI −0.028 to −0.012; *P*<.001) and the mean duration of incoming calls (95% CI −0.001 to 0.000; *P*<.001) had significant negative effects (associated with higher inhibitory control).

With regard to surrounding environments, the standard deviation of the frequency of ambient noise (95% CI −7.04e-05 to −5.83e-06; *P*=.02), the mean decibel level of ambient noise (95% CI −0.912 to −0.141; *P*=.007), the percentage of time in which the phone was connected to the internet through a mobile network (95% CI −0.027 to −0.074; *P*=.001), the Jaccard similarity coefficient for the locations visited during the past 2 hours (95% CI −0.078 to −0.005; *P*=.03), and whether an outdoor recreational place was visited during the past hour (95% CI −0.12 to −0.016; *P*=.01) had significant negative effects on predicting SSRT (associated with higher inhibitory control), whereas whether a participant was in a residential building in the past hour (95% CI 0.02-0.235; *P*=.046) and the hour before (95% CI 0.001-0.142; *P*=.045) had significant positive effects (associated with lower inhibitory control). The GEE model had an *R*^2^ score of 0.22.

### Predicting High and Low Inhibitory Control

The mean accuracy and ROC-AUC of leave-one-subject-out cross-validation were 55.4% and 57.4%, respectively, for models trained only with phone sensor features (Table 5). To further examine whether including partial data from an unseen participant can improve a model’s performance, we trained mixed models using different amounts (from 10% to 30%) of the test participant’s data. In other words, during each iteration, a certain amount of the test participant’s data along with the other participants’ data were used for training, and the model was tested on the remaining test participant’s data (which was not seen by the model during the training). The ROC-AUC slightly increased after more data from each test participant were included for training.

**Table 5:** Comparison of prediction accuracy and the area under the receiver operating characteristic curve of the different machine learning models (%).

Metrics	Model				
	Baseline^a^	LOSO^b^	Mixed (10%^c^)	Mixed (20%)	Mixed (30%)
ACC^d^	50.0	55.4	52.8	53.0	53.4
AUC-ROC^e^	50.0	57.4	57.9	58.3	60.0

^a^Model that gives predictions by chance.

^b^LOSO: leave-one-subject-out.

^c^The portion of data from the test participant used for training.

^d^ACC: accuracy.

^e^AUC-ROC: the area under the receiver operating characteristic curve.

### Features Predictive of Inhibitory Control After Including HRV Features

To include HRV features for our analysis, we first removed data points that contained missing or incomplete HRV features (details about data preprocessing are given in the *Methods* section). After data cleaning, we had a total of 577 data points. For comparison, we applied the same set of analyses as we did for phone sensor features. After removing features that had high collinearity, the features that were included in the analysis were the standard deviation of heart rate, the standard deviation of the average NN intervals during each 5-minute segment (SDANN), the root mean square of the successive differences (RMSSD), and the relative power of high frequency (HF) bands.

The predictive features after HRV features were taken into account are similar to the predictive features given by the GEE model fitted with phone sensor features. Specifically, individual inhibitory control baseline had a significant positive effect on predicting SSRT (95% CI 0.177-1.498; *P*=.01), that is, lower trait inhibitory control was associated with lower inhibitory control. Regarding activity, the standard deviation of acceleration had a significant negative effect on predicting SSRT (95% CI −0.518 to −0.052; *P*=.02), whereas the percentage of time being stationary had a significant positive effect (95% CI 0.012-0.229; *P*=.03). In other words, larger changes in one’s movements were associated with higher inhibitory control. With regard to phone use, the mean phone use duration (95% CI 1.77e-08-1.05e-07; *P*=.006) and the mean change rate of battery level when the phone was not charged (95% CI 0.005-0.164; *P*=.004) had significant positive effects on predicting SSRT (associated with lower inhibitory control), whereas the mean duration of incoming calls (95% CI −0.001 to 0.000; *P*=.007) and the number of short sessions (95% CI −0.047 to −0.025; *P*<.001) had significant negative effects (associated with higher inhibitory control). With regard to surrounding environments (also start this sentence with a new paragraph), the standard deviation of the frequency of ambient noise (95% CI −9.16e-05 to −3.01e-05; *P*<.001), the percentage of time the phone was connected to the internet through a mobile network (95% CI −0.249 to −0.039; *P*=.007), and the Jaccard coefficient of location similarity between 2 consecutive hours (95% CI −0.095 to −0.041; *P*<.001) had significant negative effects (associated with higher inhibitory control). In addition, we found that hours of sleep (95% CI −0.034 to −0.005; *P*=.008) and the standard deviation of heart rate (95% CI −7.62e-05 to 0.000; *P*=.002) also had significant effects on predicting SSRT. The estimated coefficients for the individual features are summarized in Table 6. The GEE model had an *R*^2^ score of 0.25. Similarly, we trained GBT classifiers to examine whether adding additional HRV features could improve the performance of the models. With leave-one-subject-out cross-validation, the mean AUC-ROC slightly improved to 0.62.

**Table 6:** The coefficients for all the phone sensor and heart rate variability features for predicting stop-signal reaction time estimated by the generalized estimating equation model. A positive coefficient implies that the feature is associated with higher stop-signal reaction time (lower inhibitory control).

Feature	Estimated coefficient (95% CI)	*P* value
Individual inhibitory control baseline	0.8378 (0.177 to 1.498)	.01
Hours of sleep	−0.0193 (−0.034 to −0.005)	.008
Phone use burstiness	0.0357 (0.023 to 0.048)	<.001
Mean phone use duration	6.148e-08 (1.77e-08 to 1.05e-07)	.006
Mean duration between 2 consecutive phone use sessions	8.787e-08 (5.23e-08 to 1.23e-07)	<.001
Frequency of short phone use sessions	−0.0357 (−0.047 to −0.025)	<.001
Change rate of battery level when the phone was not charged	0.0843 (0.005 to 0.164)	.04
Mean duration of incoming calls	−0.0005 (−0.001 to 0)	.007
Percentage of time being stationary	0.1205 (0.012 to 0.229)	.03
Standard deviation of acceleration	−0.2852 (−0.518 to −0.052)	.02
Standard deviation of ambient noise frequency	−6.081e-05 (−9.16e-05 to −3.01e-05)	<.001
Percentage of time connected to the internet through a mobile network	−0.1436 (−0.249 to −0.039)	.007
Jaccard coefficient of location similarity	−0.0682 (−0.095 to −0.041)	<.001
Standard deviation of heart rate	−0.0002 (−7.62e-05 to 0)	.002
Frequency of incoming calls	0.13 (−0.01 to 0.28)	.08
Frequency of outgoing calls	0.0249 (−0.023 to 0.073)	.31
Total duration of outgoing calls	−6.084e-05 (−0.001 to 0.000)	.58
Standard deviation of phone use durations	7.581e-08 (−1.72e-08 to 1.69e-07)	.11
Standard deviation of durations between 2 consecutive screen unlocks	7.451e-08 (−4.62e-08 to 1.95e-07)	.23
Frequency of charging	0.0038 (−0.037 to 0.045)	.86
Total duration of charging	−7.94e-06 (−4.78e-05 to 3.2e-05)	.70
Mean change rate of battery level when the phone was charged	0.0174 (−0.034 to 0.069)	.51
Mean decibel level of ambient noise	−0.2963 (−0.663 to 0.070)	.11
Standard deviation of the decibel level of ambient noise	0.1669 (−0.305 to 0.639)	.49
Mean RMS^a^ level of ambient noise	−0.0003 (−0.002 to 0.001)	.70
Standard deviation of the RMS level of ambient noise	−0.0001 (−0.002 to 0.001)	.87
In outdoor or recreational locations during the hour before the past hour	−0.0123 (−0.044 to 0.019)	.44
In residential buildings during the hour before the past hour	0.1663 (−0.030 to 0.363)	.10
In locations for arts or entertainment during the past hour	−0.0816 (−0.218 to 0.054)	.24
In professional or other places during the past hour	0.0688 (−0.134 to 0.271)	.51
In residential buildings during the past hour	−0.0608 (−0.234 to 0.113)	.49
SDANN^b^	−0.0003 (−0.001 to 0.000)	.32
RMSSD^c^	−0.0003 (−0.001 to 0.001)	.58
Relative power of FFT^d^ high-frequency band	0.0011 (−0.003 to 0.005)	.59

^a^RMS: root mean square.

^b^SDANN: standard deviation of the average NN.

^c^RMSSD: root mean square of the successive differences.

^d^FFT: Fast Fourier transform.

## Discussion

### Principal Findings

The aim of this study is to investigate the feasibility of using smartphones and wearable sensor data to unobtrusively assess an individual’s inhibitory control in the wild. We conducted a 4-week longitudinal study with 12 participants, collecting their sensor data continuously along with more than 1000 SST responses. We fitted GEE models with features extracted from their sensor data to analyze the main effects of the individual features and trained GBT classifiers to examine whether it is possible to classify different states of inhibitory control using these predictive features.

#### Activity

The results showed that higher levels of physical activity, which was reflected in their accelerometer data, had a positive association with an individual’s inhibitory control. This corroborates findings from the literature regarding the link between inhibitory control and exercise across different individuals [17,57]. In our study, the link between exercise and inhibitory control was also observed at an individual level. With more granular information on an individual’s physical activity, we can even potentially investigate how the intensity and the duration of physical activity impact an individual’s inhibitory control and how long the effects last in the future.

#### Phone Use

We found that certain phone use patterns were associated with changes in inhibitory control. For example,higher burstiness and longer mean intervals between 2 consecutive phone use sessions within an hour were positively associated with SSRT, which suggests that more frequent and longer phone use might be linked to a decrease in inhibitory control. However, interestingly, the number of short phone use sessions (sessions less than 30 seconds) had a positive main effect on predicting an individual’s inhibitory control, which suggests that short phone use sessions might be less detrimental than longer ones. We suspect that the short and longer phone uses might be associated with different types of phone interruptions: endogenous interruptions (sometimes referred to as internal interruptions) and exogenous interruptions (sometimes referred to as external interruptions), respectively [58]. An example of endogenous interruptions is self-initiated phone activities, such as checking one’s social media, which usually leads to a chain of other phone activities, whereas exogenous interruptions often involve phone activities resulting from external interruptions, such as receiving phone-related alerts or notifications [59,60]. Therefore, they might impact one’s inhibitory control differently.

#### Environment

The estimated coefficients for the different types of environments show that environments also play an essential role in predicting an individual’s inhibitory control. Specifically, outdoor environments, state parks for instance, had a positive association with an individual’s inhibitory control, whereas being in a residential building, such as being at home or in a dorm room, had a negative association with their inhibitory control. Moreover, the mean battery change rate when the phone was not charged and the percentage of time a user’s phone was connected to a mobile network, both of which are indicators of whether a user was outdoors, also had positive associations with their inhibitory control. Taken together, the results suggest that higher inhibitory control might be associated with people spending more time outdoors, which corroborates the findings of previous studies [19,61,62]. Future studies are needed to further examine the causal relationship between environments and inhibitory control by considering other factors, such as personality types [63].

#### HRV

According to the literature, heart rate and HRV features, particularly the relative HF power, have been found to be related to different levels of inhibitory control. We collected participants’ interbeat intervals over the course of the study. However, the standard deviation of heart rate was the only feature that was found to have a significant main effect on predicting the participants’ inhibitory control. We suspected that this may be due to some confounding factors, which were difficult to control during in-the-wild studies, such as the environment, posture, and physical activity [64]. For example, although both exercise and higher HRV contribute to higher inhibitory control, an individual’s HRV actually decreases during and after exercise due to sympathetic activation and parasympathetic withdrawal [65-67]. For future work, the information on the intensity of and the time since last physical activity will be helpful for assessing one’s inhibitory control more accurately.

#### Sleep

Studies have shown that sleep plays an essential role in inhibitory control [18]. However, in our study, sleep duration was only found to have a significant effect in predicting SSRT in the second GEE model. As hours of sleep were inferred based on a participant’s screen locks and unlocks, sometimes there might be some noise in the data (eg, a participant might wake up and check his or her phone in the middle of the night), which consequently affected the results of sleep inferencing. Future studies can combine more accurate sleep data collected through other sleep trackers, such as Oura Ring [68], along with mobile sensor data to better understand the relationship between sleep and inhibitory control.

#### Relationship Between Subjective and Objective Measurements

In this study, we employed both subjective and objective measurements to assess and compare participants’ responses for both types of measurements. We did not find any significant correlation between their subjective and objective responses. The distributions of different participants’ EMA responses were very distinct, whereas the distributions of their responses for objective measurements, SST, were relatively similar. When we looked at the correlations by location, we found that the responses for the EMA question “I am having trouble pulling myself together” had a mild positive correlation with SSRT (RMCORR=0.098; 95% CI 0.02 to 0.18; *P*=.02) in *College and University* locations. Also, in professional places (eg, office building), the responses for the EMA question “I am full of willpower” also had a slight negative correlation with SSRT (RMCORR=−0.156; 95% CI −0.31 to 0.01; *P*=.06). In other words, on campus or in the workplace, people might feel that it was harder for them to pull themselves together or had less willpower when their inhibitory control was lower. On the contrary, in outdoor or recreational places, the responses for the EMA question “I have no trouble bringing myself to do disagreeable things” had a positive correlation with SSRT (RMCORR=0.138; 95% CI 0.01-0.27; *P*=.04). In nightlife spots (eg, bars), the responses to the EMA question “I am having trouble paying attention” had a marginal negative correlation with SSRT (RMCORR=−0.216; 95% CI −0.44 to 0.03; *P*=.09).

The little correlation between subjective and objective measurements was also observed in some recent studies. Some plausible explanations for the inconsistency suggested by the researchers include (1) questions in self-report scales may capture multiple constructs rather than inhibitory control alone, whereas inhibitory control tasks typically measure a narrower construct; (2) behavioral inhibition tasks, including SSTs, were designed to minimize between-subject variability to achieve high reliability (which corroborates our findings that the distributions of participants’ SSRTs were fairly similar), a phenomenon called reliability paradox, which limits the tasks’ ability to capture individual differences; (3) what inhibitory control tasks measure might be participants’ *maximum* rather than their *actual* inhibitory control at that moment, as participants had been instructed to perform as accurately as possible; (4) there might be some publication bias in the earlier literature that overestimated the correlation between self-reports and inhibitory control tasks [69-71]. In addition to these explanations, we also suspect that there might be some discrepancy between the perceived level of inhibitory control and the actual level of their inhibitory control. In other words, people might not be aware of their diminished inhibitory control and still report high inhibitory control. Further, their self-reported inhibitory control might be biased by the level of their inhibitory control at the moment [31]. The gap between one’s perceived and actual inhibitory control might depend on different contexts, such as locations. Future studies that combine both automatic and manual location logging will allow collection of more accurate information on semantic locations and help examine how different types of locations influence the way people perceive and self-report their inhibitory control.

In summary, we identified several markers that can be used to infer inhibitory control in the wild. However, these are only a subset of markers that manifest the effects of the factors modulating an individual's inhibitory control. There is information on other factors, such as diet, stimulant intake, and exposure to natural light, which is difficult to directly and accurately measure using smartphone sensors, which in turn limits the performance of our prediction models. That said, being able to track patterns that are associated with inhibitory control is still of great use in many real-world applications, such as productivity management technologies. Showing statistics about users’ phone use patterns, physical activities, location patterns, and inferred levels of inhibitory control during the past few weeks can provide insights into how their inhibitory control fluctuates over time. As such, users can adjust their behaviors to change the pattern of their inhibitory control. Alternatively, they can schedule their daily tasks according to their inhibitory control patterns so that they work on tasks requiring a great level of attention when they are in states of high inhibitory control.

### Potential Use Scenarios

#### Workplace Productivity and Well-Being

Previous studies have shown that there are contexts in the workplace where people are more susceptible to distractions. For example, people are more likely to cyberloaf after returning from a break [72]. Therefore, some intervention designs have been proposed to help mitigate the proneness to distractors [73,74]. The triggering moments of the interventions are usually rule based, for instance, the first 15 min after a physical break or pomodoro technique. However, the amount of time it takes for an individual to get to a focused state, namely, a high inhibitory control state, might vary from person to person depending on factors such as the environment and the type of work. If we can integrate the technology of unobtrusive inhibitory control assessment into those intervention technologies, it will allow such intervention technologies to more accurately assess an individual’s focus state and provide more personalized interventions based on that information. For example, an intervention system can infer the level of a user’s inhibitory control based on their phone use patterns, such as patterns indicating self-interrupts, and restrict their access to contents or applications that might render them vulnerable to a chain of distractions, or even delay the arrival of notifications, to keep them stay focused.

#### Addictions

There has been growing awareness of digital well-being and phone addiction. Android and iOS have included screen time to help users restrain the amount of time they spend on their phones and prevent excessive phone use. However, prolonged phone use time is just one of the behaviors [75] that indicates addictive phone use. Other behaviors, such as frequently checking the phone, might also be indicators of excessive phone use. As phone addiction is highly related to the level of an individual’s inhibitory control [76], tracking a person’s inhibitory control continuously and prompting them to take preemptive steps when they have low inhibitory control will be more effective in preventing phone addiction.

In addition to excessive phone use, there are other types of addictive behaviors, such as food addiction and substance dependence, which also result from reduced inhibitory control. Detecting the downward trends in those individuals’ inhibitory control and the signatures and contexts that are associated with the decrease in their inhibitory control can prevent them from falling off the wagon by suggesting that they take some preemptive steps. For example, a system can track whether a user is in an environment that is likely to trigger alcohol craving based on the type of location and the loudness in the surrounding environment [77] and suggest that the person take an alternative route when he or she is in proximity to that type of environment.

#### Mental Disorders and Illnesses

The level of inhibitory control is also related to which episode an individual with mental disorders is in. For example, studies have shown that patients with bipolar disorder had impaired inhibitory control when they were in their manic and depressive episodes [78]. As such, a system that is able to continuously track the level of a bipolar patient’s inhibitory control can potentially help detect the early signs of mood swings and provide timely interventions.

### Limitations and Future Work

The size of our study was relatively small and homogeneous (the participants were mainly recruited from a college campus and were limited to iOS users). Follow-up studies with a large sample size and more diverse demographics will need to be conducted to examine how different demographic factors impact inhibitory control. Other information, such as light and app use, which we hypothesized would be useful for inferring inhibitory control, could not be collected on iOS devices (because the majority of the participants on campus were iOS users). The phone use patterns of users who installed productivity apps might be different from the phone use patterns of those who did not. Besides, outliers (of SST performance and inferred sleep) were removed from our analysis. Further research is needed to investigate whether such extreme values will affect the assessment of inhibitory control. For future work, in addition to phone sensor and heart rate data, we are planning to collect data using other types of wearables, such as fitness or sleep trackers, to obtain more granular information, such as physical activity, sleep quality, and chronotype, to account for the influences of these confounding factors. Being able to account for these pieces of information can potentially improve models’ performance for inferring different states of inhibitory control.

### Conclusions

This paper presents a preliminary study using mobile and wearable sensor data to assess individuals’ inhibitory control in the wild. To the best of our knowledge, this is the first in-the-wild study aimed to develop a new approach to accessing inhibitory control unobtrusively and continually. On the basis of the data from 12 participants during a 4-week study, we found that factors including an individual’s inhibitory control baseline, the number of phone use sessions, the change rate of battery level, the mean duration of incoming calls, physical activity, the mean frequency and loudness of ambient noise, whether a user was outdoor, and the types of locations a user visited had significant main effects on predicting an individual’s inhibitory control. We also trained a machine learning model to predict whether an individual had high or low inhibitory control at different moments using those features, which achieved an AUC-ROC of 60%. The findings provide some insights into how we can potentially design personalized technologies that can help support and manage productivity in school or the workplace and intervention tools that can prevent individuals with mental disorders such as substance abuse from relapsing or engaging in problematic behaviors.

## Abbreviations

API: application programming interface
AUC-ROC: area under the receiver operating characteristic curve
BIS: Barratt Impulsiveness Scale
EMA: ecological momentary assessment
GBT: gradient boosting tree
GEE: generalized estimating equation
HF: high frequency
HRV: heart rate variability
RMCORR: repeated measures correlation.
SSD: stop-signal delay
SSID: service set identifier
SSRT: stop-signal reaction time
SST: stop-signal task
